# Robust Estimation of Contact Force and Location for Magnetic-Field-Based Soft Tactile Sensor Considering Magnetic Source Inconsistency

**DOI:** 10.3390/s21165388

**Published:** 2021-08-10

**Authors:** Xiaofeng Yang, Bingchu Li, Lihong Yang, Huimin Shen

**Affiliations:** School of Mechanical Engineering, University of Shanghai for Science and Technology, Shanghai 200093, China; 192351375@st.usst.edu.cn (X.Y.); lhyang@usst.edu.cn (L.Y.); hmshen@usst.edu.cn (H.S.)

**Keywords:** flexible tactile sensor, magnetic source tracking, robust estimation

## Abstract

Flexible magnetic-field-based tactile sensors (FMFTS) have numerous advantages including low cost, ease of manufacture, simple wiring, high sensitivity, and so on. Flexible magnetic-field-based tactile sensors need to be calibrated before use to build accurate mapping between contact force and magnetic field intensity measured by magnetic sensors; however, when considering remanence inconsistency of magnetic source, each FMFTS needs to be calibrated independently to enhance accuracy, and the complex preparation prevents FMFTS from being used conveniently. A robust estimation method of contact force and location that can tolerate remanence inconsistency of magnetic source in FMFTS is proposed. Firstly, the position and orientation of magnetic source were tracked using the Levenberg–Marquart algorithm, and the tracking results were insensitive to the remanence of magnetic source with appropriate cost function. Secondly, the mapping between magnitude and location of contact force and position and orientation of magnetic source was built with calibration of one sensor; the mapping only depends on the structural response of flexible substrate, and thus can be extended to estimate external force and location for other sensors with the same structure. The proposed method was evaluated in both simulations and experiments, and the results confirm that the estimation of magnitude and location of external force for FMFTS with the same structure and different remanence could reach acceptable accuracy, depending on single calibration. The proposed method can be used to simplify the calibration procedure and remove the barrier for large-scale application of FMFTS and replacement of damaged FMFTS.

## 1. Introduction

Tactile sensors made of flexible materials can better adapt to the external environment and provide more information, such as contact pressure, contact location, the texture characteristics, local shape, and material characteristics of the object [1]. The development of tactile sensors has great potential for wearable devices [2], biomedical systems [3], human–computer interaction systems [4], and intelligent robots [5], and promotes the development of intelligent systems [6].

At present, flexible tactile sensors draw from the following measuring principle, including capacitive [7], resistive [8], piezoelectric [9], optical [10], magnetic [11], vision-based [12,13], etc. The tactile sensors based on capacitive, resistive, and piezoelectric were generally combined in array to expand the sensing area; the pitch of the taxel (tactile pixel) limits the spatial resolution of such tactile sensors. Although the miniaturization of sensing unit could improve resolution, this means complex manufacturing and wiring. Vision-based tactile sensors, such as GelSight [12] and DIGIT [13], can achieve high resolution; however, this brings higher cost and limits of installation space due to the use of cameras. By comparison, FMFTS [14,15,16,17] embodies some wonderful features including less wiring, low cost, and high resolution. FMFTS is generally constituted of three parts: magnetic source (permanent magnet or magnetic powder), flexible substrate, and magnetic sensors. The shape of the flexible substrate could be rectangular solid, cylindrical, pyramid, etc., each shape shows different mechanical responses. When the contact force is applied to the surface of the flexible substrate of FMFTS, the position and orientation (pose) of permanent magnets embedded in the flexible substrate or the distribution of the magnetic powder will change, and the magnetic field distribution varies accordingly, which could be captured by magnetic field sensors [18] through magnetic flux density data monitoring. FMFTS have received considerable attention in recent years.

Force and location estimation was necessary for FMFTS, which depends on the relationship between external contact force and magnetic field distribution. The nonlinear relationship between the magnetic flux density and the contact force can be established through analytical models [19] or calibration; both look-up tables (LUP) [20] and neural networks [21] have been used to solve the inverse problem to acquire the external force (normal or tangential) [22]. Chathuranga et al. used spring theory and bending theory to build the analytical model of a flexible three-axis force sensor [19], however, the analytical modeling method was hard to apply to wide sensors considering complex structure responses of FMFTS. Dwivedi et al. used neural networks to build the mapping between three-dimensional external force and magnetic flux density, and then estimated the normal force and tangential force; however, the contact location was neglected [14]. FMFTS array was used for distributed sensing. Tomo et al. developed a permanent magnet-based tactile sensors array which contains 16 tri-axial taxels, each unit consisting of a hall sensor, and a magnet which was used to measure the three dimensional force [23]. The sensing elements were discrete in the array and the distance between sensing elements limited the resolution of this kind of sensor. Hellebrekers et al. developed a soft magnetic tactile skin for continuous force and position estimation; they mixed magnetic particles in a flexible medium and built the relationship between contact force and magnetic flux density through complex calibration: neural network was used to estimate the external force and location [21]. Nevertheless, since the distribution of magnetic particles in the flexible substrate is uncertain, it is necessary to recalibrate and train the neural network when making a new soft magnetic tactile sensor. Mohammadi et al. proposed a permanent magnet-based soft tactile sensor with a continuous medium, and used the K-nearest neighbors classification algorithm to calculate the location of the contact force [24], but the magnitude of contact force was unavailable.

Previous studies on force and location estimation method are mostly based on establishing the mapping between contact force and magnetic field through calibration. The calibration relies on large volumes of tests with different magnitude and location of contact force and is time consuming. For replacing damaged sensors [25], or large-scale use of FMFTS, the convenience of calibration for a large number of sensors must be considered, as it is expected that the calibration results of one sensor could be applied to sensors with same specification. The generality of calibration results depends on the consistency of magnetic source and flexible substrate between different FMFTS; however, magnetization error of magnets, demagnetization of magnets, and molding deviation of flexible substrate is unavoidable in mass production and use of FMFTS. In face of magnetization error or demagnetization, the remanence of each permanent magnet in FMFTS may be different, the calibration results can no longer directly used in new tactile sensors, and recalibration is needed. Compared with magnetization error and demagnetization, the molding deviation of flexible substrate has subtle influence on force and location estimation at current manufacturing accuracy; when the same contact force is applied to FMFTS, the variation of position and orientation of the permanent magnet is approximately the same between different sensors. Inspired by permanent magnet-based localization [26], if one can convert the magnetic field information into the pose of permanent magnet, the influence of magnetic source inconsistency could be reduced, and recalibration can be omitted.

In this paper, a robust force and location estimation method for FMFTS, which could ensure accuracy and convenience of tactile sensors considering magnetic source inconsistency, is proposed. Compared with existing estimation methods, the proposed method can be separated into two parts: pose estimation for permanent magnet, and reasoning of external force based on pose of the permanent magnet. The magnetic source tracking algorithm can estimate the pose of permanent magnet based on magnetic flux density, and then the reasoning of external force will conduct based on the mapping between contact force and pose of permanent magnet. The calibration in the proposed method aims to build mapping between contact force and pose of permanent magnet, rather than mapping between contact force and magnetic flux density in the traditional force and location estimation method. Considering that the mapping between contact force and pose of permanent magnet depends on structure response of substrate, the calibration process can thus be simplified for sensors with identical mechanical structure and material properties. Using the proposed method, the calibration result in one sensor can be extended to estimate the external contact force for new sensors with the same mechanical structure and material properties. The innovation of this paper is a new force and location estimation mechanism that decouples the pose estimation of permanent magnet from force and location estimation for FMFTS, which could simplify the calibration process and provides convenience for mass use of FMFTS. Furthermore, in case the flexible substrate of FMFTS is damaged or the permanent magnet is demagnetized, sensors produced in the same batch can be directly used to replace the broken one without recalibration.

The subsequent sections are arranged as follows: Section 2 describes the scheme and principle of robust force and location estimation method for FMFTS; the proposed method is evaluated in Section 3 using datasets from finite element simulation; Section 4 presents the experiment to verify the proposed method; Section 5 draws conclusions and presents future work.

## 2. Materials and Methods Robust Force and Location Estimation Method

### 2.1. Scheme of the Proposed Method

**Figure 1:**
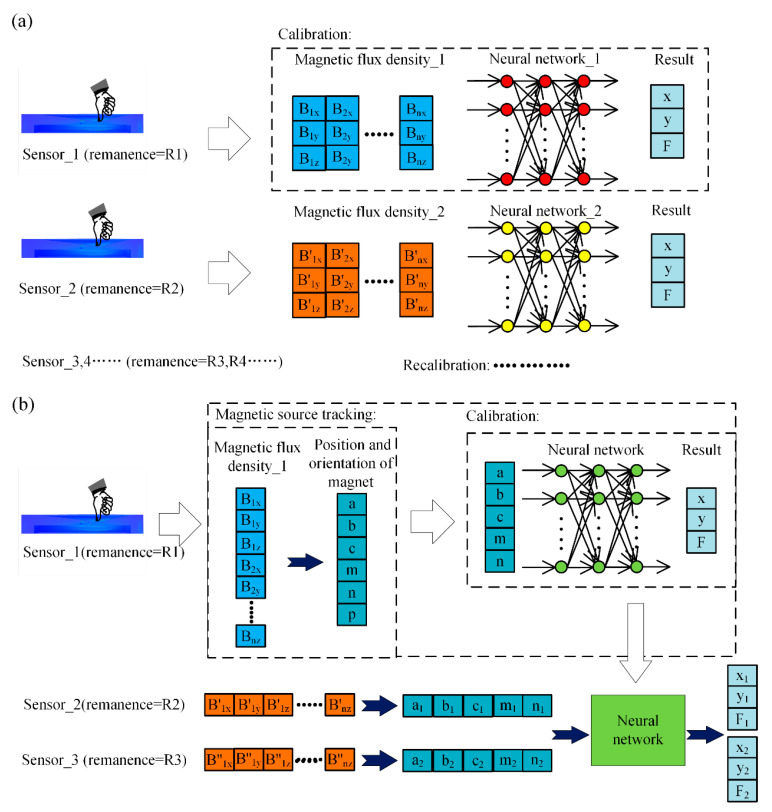
(**a**) Contact force and location estimation in previous research; (**b**) robust contact force and location estimation method in this paper.

In this paper, the poses of the permanent magnet embedded in FMFTS are solved by magnetic source tracking algorithm, and then the relationship between the contact force and location and the pose of the permanent magnet is established. The method to calculate contact force and location uses the diffusion effect of flexible substrate, when external force is applied to the surface of flexible substrate, the pose of the permanent magnet embedded in the flexible substrate will change. It is possible to determine unknown external force and location according to movement of permanent magnet in FMFTS. The previous research directly constructs the relationship between the magnetic flux density induced by permanent magnet and external force, as shown in Figure 1a. The relationship between the magnetic flux density and external force in different FMFTS may be very different due to magnetic source inconsistency. As a result, new tactile sensors need to be recalibrated; in other words, the invalidity of previous calibration results was mainly due to remanence inconsistency. For tactile sensors manufactured in same batch, the response of permanent magnet’s position and orientation under the action of contact force is approximately the same. The method proposed in this paper is shown in Figure 1b, and the proposed method can be separated into two parts: pose estimation for permanent magnet, and reasoning of external force based on pose of permanent magnet. The main difference between the proposed method and previous methods is to decouple pose estimation for permanent magnet from force and location estimation of FMFTS. The magnetic source tracking algorithm can estimate the pose of the permanent magnet in the face of remanence inconsistency; thus, the robustness of the force and location estimation for FMFTS can be improved and calibrations can be simplified by the magnetic source tracking algorithm.

**Figure 2:**
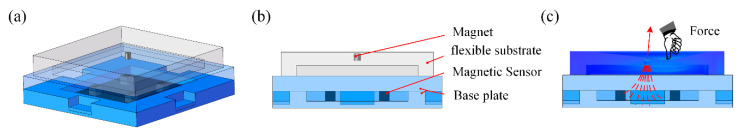
(**a**) Concept design of FMFTS; (**b**) components of the FMFTS; (**c**) deformation of FMFTS and variation of magnetic flux density caused by external force.

The structure of FMFTS selected in this article is shown in Figure 2, and the structure of the flexible substrate can be changed for different application scenarios.

The method is described in the steps below: 1. Construct the objective function for magnetic source tracking algorithm; 2. Solve the optimization problem using LM method to estimate permanent magnet’s pose; 3. The calibration points are collected on FMFTS sample (Sensor_1), magnetic flux densities are captured under external force with different magnitude and location, magnetic flux densities are converted into pose of permanent magnet, and neural network is trained to construct the relationship between contact force and location and pose of permanent magnet; 4. For unknown contact force acts on Sensor_1 and other FMFTS (Sensor_2), the magnetic flux densities captured by magnetic sensors are converted into pose of permanent magnet, with the pose information as inputs of the trained neural network, and the contact force and location as outputs.

### 2.2. Magnetic Source Tracking Algorithm

**Figure 3:**
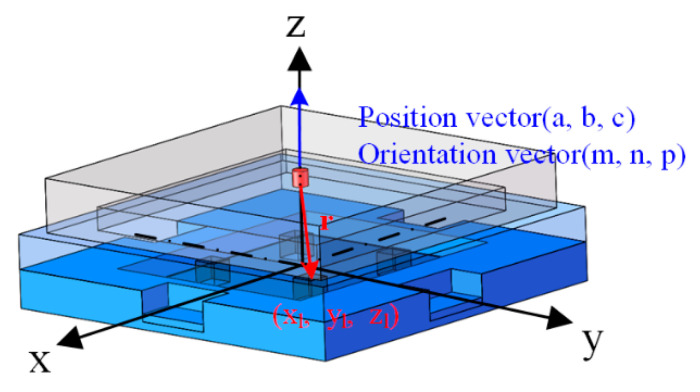
The magnetic dipole model: (a,b,c),(m,n,p) are the position vector and orientation vector of the permanent magnet in space; the location of the l th magnetic sensor is $\left(x_{l},y_{l},z_{l}\right)$; r is the vector pointing from the center of the permanent magnet to the magnetic sensor.

The pose estimation of permanent magnet embedded in FMFTS needs to establish its magnetic field distribution model. In this paper, the axial magnetized cylindrical permanent magnet that most FMFTS adopt is used, and the permanent magnet is approximated as the magnetic dipole model [27]. The magnetic field distribution model is shown in Figure 3.

The radius of cylindrical permanent magnet is $r_{mag}$, the length is L, and the residual magnetization is M. The magnetic flux density generated by the permanent magnet at any point in space can be expressed as [28]:

(1) $$\vec{B}=\frac{\mu_{0}}{4\pi }\left[\frac{3(\vec{m}•\vec{r})\vec{r}}{r^{5}}-\frac{\vec{m}}{r^{3}}\right]$$

where $\mu_{0}$ is the vacuum permeability, $\vec{m}$ is the magnetic dipole moment of permanent magnet, which can be expressed as $\vec{m}=V\vec{M}=(\pi r_{mag}^{2}L)(M\hat{m})$, and $\hat{m}$ is the unit vector of the direction of the magnetic dipole moment. Therefore, Equation (1) can be further expressed as:

(2) $$\vec{B}=\frac{\mu_{0}r_{mag}^{2}LM}{4}\left[\frac{3(\hat{m}•\vec{r})\vec{r}}{r^{5}}-\frac{\hat{m}}{r^{3}}\right]=B_{T}\left[\frac{3(\hat{m}•\vec{r})\vec{r}}{r^{5}}-\frac{\hat{m}}{r^{3}}\right]$$

where $B_{T}$ is related to the characteristics of permanent magnet, and the remaining part is related to the pose of the permanent magnet. The magnetic flux density at the location of the lth magnetic sensor is:

(3) $${\vec{B}}_{l}=B_{lx}\vec{i}+B_{ly}\vec{j}+B_{lz}\vec{k}\;(l=1,2,\cdots \cdots ,N)$$

where $B_{lx}$, $B_{ly}$, $B_{lz}$ could be represented by

(4) $$B_{lx}=B_{T}\left\{\frac{3\left[m(x_{l}-a)+n(y_{l}-b)+p(z_{l}-c)\right](x_{l}-a)}{R_{l}^{5}}-\frac{m}{R_{l}^{3}}\right\}$$

(5) $$B_{ly}=B_{T}\left\{\frac{3\left[m(x_{l}-a)+n(y_{l}-b)+p(z_{l}-c)\right](y_{l}-b)}{R_{l}^{5}}-\frac{n}{R_{l}^{3}}\right\}$$

(6) $$B_{lz}=B_{T}\left\{\frac{3\left[m(x_{l}-a)+n(y_{l}-b)+p(z_{l}-c)\right](z_{l}-c)}{R_{l}^{5}}-\frac{p}{R_{l}^{3}}\right\}$$

where $R_{l}=\sqrt{{\left(x_{l}-a\right)}^{2}+{\left(y_{l}-b\right)}^{2}+{\left(z_{l}-c\right)}^{2}}$.

When the cylindrical permanent magnet rotates around its central axis, the magnetic field around it does not change with the rotation of the magnet, so the following constraints are added:

(7) $$m^{2}+n^{2}+p^{2}=1$$

In this paper, four three-axis magnetic sensors were used in one FMFTS. From the equation system composed of Equations (4)–(7), it can be seen that there are five unknown parameters, twelve equations need to be solved to acquire all unknown parameters, and all equations are statically indeterminate nonlinear. It is difficult to solve such equations analytically, therefore nonlinear numerical optimization methods were generally used.

To use the numerical optimization method, it is necessary to construct the objective function. According to Formulas (4)–(6), the theoretical magnetic field value of the permanent magnet at the lth three-axis magnetic sensor can be obtained, and the actual measured magnetic flux densities can be obtained through communications with magnetic sensors. The objective function was given by finding the difference between the theoretical value and the measured value of magnetic sensors, as follows:

(8) $$E_{i}=\sum_{l=1}^{N}\left(B_{li}^{\prime }-B_{li}\right)$$

where i represents space axis (x, y or z), and $B_{li}^{\prime }$ and $B_{li}$ are the measured value and theoretical value of lth magnetic sensor in axis i.

(9) $$x=arg\;min\;(E_{x}^{2}+E_{y}^{2}+E_{z}^{2})$$

where x=(a,b,c,m,n,p).

It can be seen from Equation (9) that this is a nonlinear least squares problem. There are many methods to solve this kind of problems. The Levenberg–Marquardt (LM) method is a frequently used method for solving nonlinear least squares. It combines the advantages of the Gauss–Newton method and steepest descent method. The iterative formula is as follows:

(10) $$x_{k+1}=x_{k}-{\left(J^{T}J+\mu I\right)}^{-1}g_{k}$$

where $J^{T}J$ is approximate value of Hessian matrix, $g_{k}$ is negative gradient direction, I is identity matrix. μ is damping coefficient, under the condition that μ is greater than zero, the matrix can be guaranteed to be positive definite. When μ is very large, the iteration process is close to the steepest descent method, and when μ is small, it is close to the Gauss–Newton method. Previous studies have proved that this method has higher positioning accuracy [28]. Therefore, this paper selects the LM algorithm to solve the pose of the permanent magnet. The initial point of the optimization algorithm in this paper has been given, namely the pose of the permanent magnet in the unstressed state of the tactile sensor.

### 2.3. Reasoning of External Force Based on Position and Orientation of Magnet

The pose of the permanent magnet under different contact forces and location can be obtained using LM algorithm, and then the relationship between the pose of the permanent magnet and contact force and location can be established. The relationship is nonlinear due to the characteristics of the structure and material of flexible substrate. Given any pose of permanent magnet, reasoning of external force aims to find the potential external force that leads to the corresponding pose. According to the mapping between pose of the permanent magnet and external contact force and location, neural networks have been proven to be an efficient solution, and are not limited by the nonlinear model of flexible substrate [29]. Back propagation (BP) neural network and radial basis function (RBF) neural network are the most widely used neural networks in practical applications and can approximate any nonlinear function with reasonable precision. In this paper, in order to compare the effectiveness of different neural networks, the mapping relationships between the pose of permanent magnet and external contact force and location were established using BP neural network and RBF neural network.

**Figure 4:**
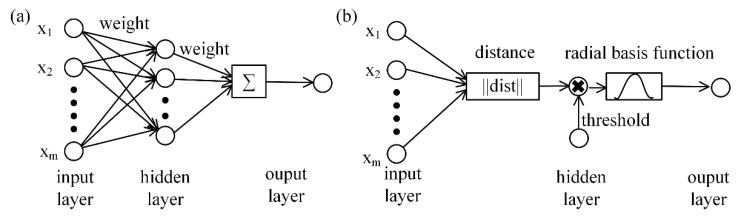
Structure of neural networks. (**a**) BP neural network; (**b**) RBF neural network.

The structures of BP neural network and RBF neural network are shown in Figure 4. BP neural network works on the idea of error back propagation, which continuously corrects the weights and thresholds in the network, so that the error between the expected output and the predicted value is continuously reduced until the accuracy requirements are met. The RBF neural network uses the radial basis function to form a hidden layer and maps the input vector from low-dimensional to high-dimensional without weight connection, so that the low-dimensional linear inseparability can be separated in high-dimensional. In this paper, the MATLAB neural network toolbox is used to establish the mapping relationship between the pose of permanent magnet and external force and location.

## 3. Evaluation of Robust Force and Location Estimation Method with Simulation

To evaluate the robustness of the proposed estimation method in the face of remanence inconsistency, firstly, the magnetic field induced by permanent magnet is analyzed numerically, and magnetic fields for permanent magnets with the same pose and different remanence are compared. Secondly, the pose estimation of permanent is evaluated; the magnetic field distributions of permanent magnets with the same poses and different remanence are different, however, using the pose estimation method, the pose of permanent magnets with different remanence can be correctly estimated. Third, the magnetic–solid coupling model of FMFTS is established; the magnetic flux densities for sensors with different remanence under various contact force and locations are collected, upon which the proposed force and location estimation method are trained and evaluated.

### 3.1. Simulation of Magnetic Field of the Permanent Magnet

**Figure 5:**
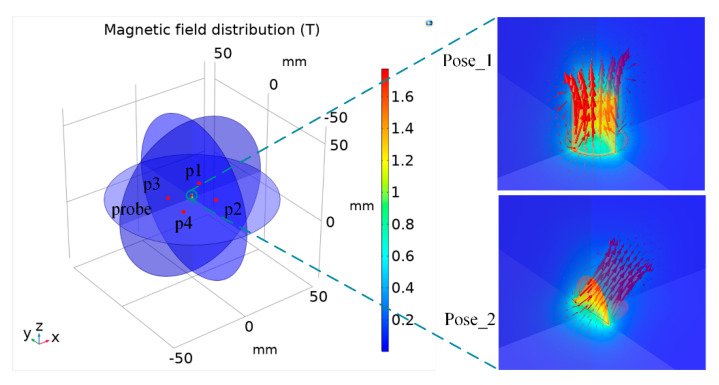
Magnetic field simulation model of the permanent magnets in two poses.

In this section, the finite element model of the permanent magnet is firstly established in COMSOL 5.5 (COMSOL Inc., Stockholm, Sweden). The size of the permanent magnet is set to 2 mm in diameter, 2 mm in height, and the material is NdFeB. Taking a sphere with the radius of 50 mm around the permanent magnet as the air model, the outer surface of the sphere is selected as the zero magnetic scalar potential surface and used as the initial condition for applying the magnetic field. In order to obtain the magnetic flux density in the space, domain point probes were set at four points, namely p1 (8, 8, 0), p2 (8, −8, 0), p3 (−8, 8, 0), and p4 (−8, −8, 0), equivalent to four magnetic sensors, as is shown in Figure 5.

According to magnetization error, the remanence difference between permanent magnets of the same grade is about 0–0.05 T [30]. The position and orientation of permanent magnet can be expressed as a vector (a, b, c, m, n, p), where (a, b, c) represents the three-axis position coordinates, respectively, and (m, n, p) represents the direction vector. In the simulation, the first position and orientation (Pose_1) of the permanent magnet is set to (0, 0, 12, 0, 0, 1), the remanence of the permanent magnet is set to 1.18 T, 1.20 T, 1.23 T, 1.25 T, 1.28 T, respectively, and then the three-axis magnetic density at the four probes is obtained. The second position and orientation (Pose_2) of the permanent magnet is set to (0.707, 0, 11.707, 0.707, 0, 0.707) and the above steps repeated. The results are shown in Table 1.

### 3.2. Position and Orientation Tracking of Magnet

To deal with the magnetic field information collected by magnetic sensors in simulation, we established the objective function and LM algorithm in MATLAB2016 (Mathworks, Natick, MA, USA), and the pose of the permanent magnet can be estimated. In Equations (2) and (9), the remanence of permanent magnet is theoretically set to 1.23 T, which means the target remanence without magnetization error. The pose estimation results are shown in Table 2.

It can be seen from Table 1 and Table 2 that when the permanent magnets are in the same pose (Pose_1 or Pose_2), the magnetic field distribution of permanent magnets with different remanence are different. After the permanent magnet’s pose is solved by the LM algorithm, the correct pose is estimated in the face of remanence inconsistency. The Pose_1 is theoretically (0, 0, 12, 0, 0, 1), and poses of the permanent magnets with different remanence obtained by the LM algorithm are approximately (−0.0313, 0.0443, 12.2578, −0.0052, 0.0051, 1.0232). The case of Pose_2 is similar. Therefore, when the mapping between force and location and pose of the permanent magnet is constructed, other sensors can directly use this mapping to predict contact information.

### 3.3. Force and Location Estimation

The FMFTS works on the coupling between magnetic field of the permanent magnet and deformation of the flexible substrate. In this section, we establish the coupling model for solid mechanics and magnetic field to simulate the actual response of FMFTS with external contact force.

#### 3.3.1. Magnetic–Solid Coupling Model

In this paper, the polydimethylsiloxane (PDMS) is selected as the substrate. It is a hyperelastic material, and the relationship between stress and strain is nonlinear. The Neo-Hookean model, Ogden model, and Mooney–Rivlin two-parameter model are commonly used hyperelastic material models [31]. Since the Mooney–Rivlin two-parameter model can better express the hyperelastic properties of PDMS when the material is with small strain, we choose the Mooney–Rivlin two-parameter model for finite element analysis. The expression of strain energy density of the Mooney–Rivlin two-parameter model is:

(11) $$W=C_{10}(I_{1}-3)+C_{01}(I_{2}-3)+\frac{1}{d}{\left(J-1\right)}^{2}$$

where W is the strain potential energy function, $C_{10}$ and $C_{01}$ are the material constants, $I_{1}$ and $I_{2}$ are the first strain deviation invariant and the second strain deviation invariant respectively, d is the material incompressible parameter, and J is the relevant parameter.

(12) $$C_{10}=\frac{E}{5(1+v)}\;C_{01}=\frac{E}{20(1+v)}$$

where E is the elastic modulus of PDMS, and v is the Poisson’s ratio of PDMS. The initial bulk modulus is defined as

(13) $$K=\frac{2}{d}$$

where $d=\frac{1-2v}{C_{10}+C_{01}}$.

**Figure 6:**
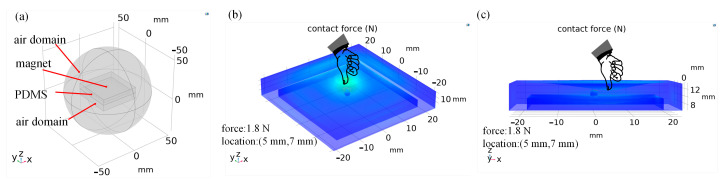
Flexible magnetic-based tactile sensor simulation model: (**a**) components of the model; (**b**,**c**) the simulation results of force applied to FMFTS with the magnitude of 1.8 N and the location of (5 mm, 7 mm).

PDMS can change the elastic modulus according to different ratios of its basic components and curing agent [32], In the simulation, set E = 1.21 MPa, v = 0.49, $C_{10}$ = 0.162416 MPa, $C_{01}$ = 0.040604 MPa, and K = 20.30201 MPa. The magnetic–solid coupling simulation model for FMFTS is shown in Figure 6; the degree of mesh refinement has a great influence on simulation accuracy, the spherical air domain is divided into finer mesh, and the grid at the position of probes is extremely fine.

The range of the tactile sensor can be considered as the force corresponding to the maximum displacement at the center of the sensor; if the contact force continues to increase, FMFTS will present different performance due to contact with the base plate, so the range is set to 2.0358 N in this simulation. The minimum external force can be considered as the force corresponding to the minimum change of magnetic flux at the boundary that can be detected, which is set to 2.827 mN. It is noteworthy that the maximum and minimum external force for FMFTS in this simulation are affected by elastic modulus and thicknesses of the PDMS.

The magnetic flux densities under remanence of 1.23 T and 1.28 T, and contact force of 0.2827 N, 1.1310 N, and 1.9792 N, are collected. The action area of contact force is a circular area with diameter of 6 mm on the surface of FMFTS; within such area, forces are applied every 2 mm and the magnetic flux densities on four probes are recorded. A total of 675 datasets were collected under remanence 1.23 T; 675 datasets were used for calibrating and 90 datasets were used for testing. A total of 90 datasets were collected under remanence of 1.28 T; such datasets were used to verify whether the calibration results constructed under remanence of 1.23 T can be used for sensors with remanence of 1.28 T.

#### 3.3.2. Force and Location Estimation Results

Under the forces of 0.2827 N, 1.1310 N, and 1.9792 N at different contact locations, the three-axis magnetic flux densities at the four probes are generated, and the pose of the permanent magnet under different forces can be obtained through pose estimation. We use the data collected by the FMFTS with a remanence of 1.23 T for training to construct the mapping between contact force and location and pose of permanent magnet; the mapping between contact force and location and magnetic flux densities are also built for comparison. Then, the test datasets collected by FMFTS with remanence 1.23 T and remanence 1.28 T were used to verify the accuracy of force and location estimation.

In MATLAB, the BP model and RBF model are established by using the newff function and the newrb function. The BP neural network parameters include hidden layer size, activation function, and training function. The RBF neural network parameters include spread of radial basis functions, maximum number of neurons, and all parameters are selected through five-fold cross-validation.

The force and location estimation was evaluated in four cases:

Case 1: The training sets are acquired on FMFTS which has embedded permanent magnet of remanence 1.23 T; BP neural network was built through calibration to represent the mapping between contact force and location and magnetic flux densities, namely BP_ftp_1.23.

Case 2: The training sets are acquired on FMFTS which has embedded permanent magnet of remanence 1.23 T; BP neural network was built through calibration to represent the mapping between contact force and location and pose of the permanent magnet, namely BP_ptp_1.23.

Case 3: The training sets are acquired on FMFTS which has embedded permanent magnet of remanence 1.23 T; RBF neural network was built through calibration to represent the mapping between contact force and location and magnetic flux densities, namely RBF_ftp_1.23.

Case 4: The training sets are acquired on FMFTS which has embedded permanent magnet of remanence 1.23 T; RBF neural network was built through calibration to represent the mapping between contact force and location and pose of the permanent magnet, namely RBF_ptp_1.23.

**Figure 7:**
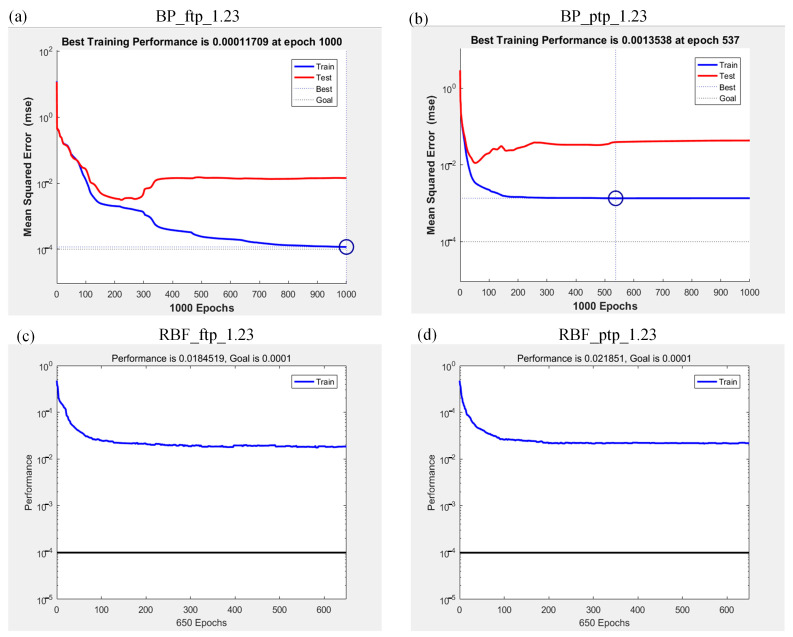
Performance of neural network: (**a**) case 1; (**b**) case 2; (**c**) case 3; (**d**) case 4.

The neural network’s performance of the four cases is shown in Figure 7. As can be seen from Figure 7, the performance of BP_ftp_1.23, BP_ptp_1.23, RBF_ftp_1.23, and RBF_ptp_1.23 are 0.00011709, 0.0013538, 0.0184519, and 0.021851, respectively. The performance of BP_ftp_1.23 is slightly better than BP_ptp_1.23, and RBF_ftp_1.23 is also slightly better than RBF_ptp_1.23, which can be explained by the fact that the pose estimation of permanent magnet is an optimization process and causes discontinuities between the train data, and the RBF neural network does not perform as well as the BP neural network in this scenario (note: “Test” in Figure 7a,b represents a part of the data extracted from the training set to verify the performance of the network, and the specific test results are as follows).

**Figure 8:**
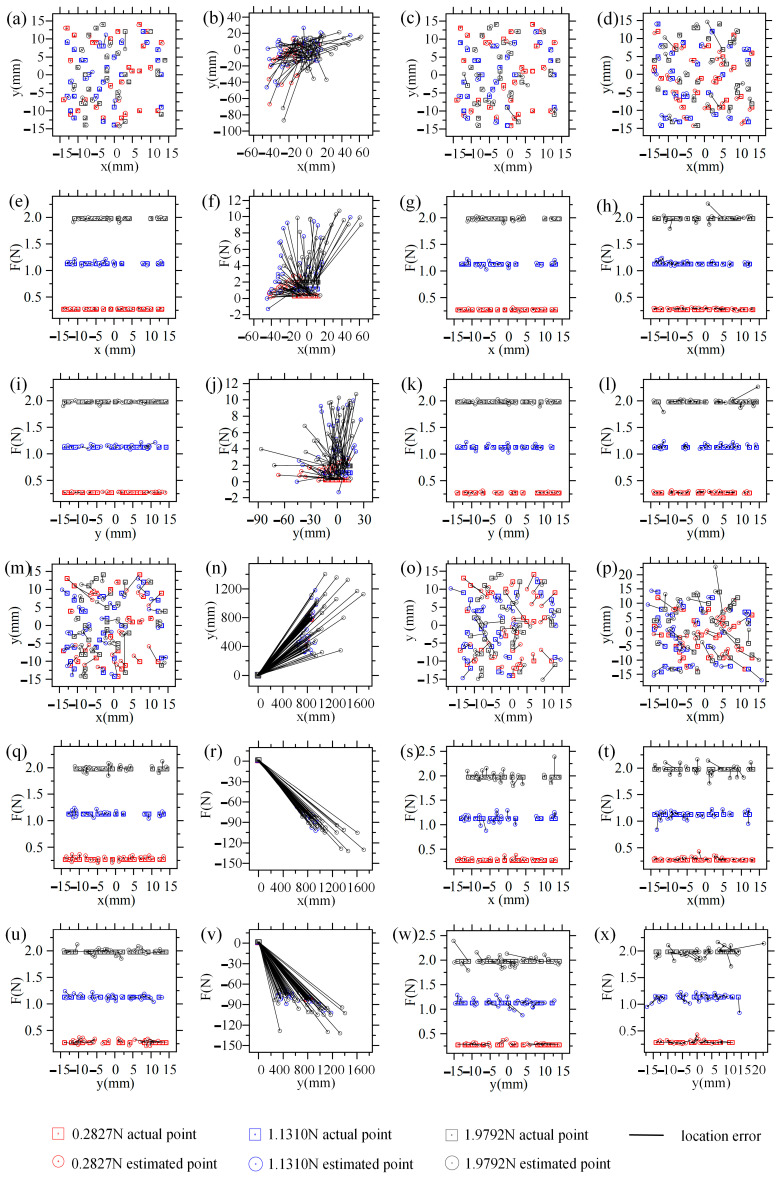
Force and location estimation results: (**a**–**l**) are the estimation results of BP neural network; (**m**–**x**) are the estimation results of RBF neural network; (**a**,**b**,**e**,**f**,**i**,**j**) case 1, (**a**) actual locations and estimated locations based on test datasets from FMFTS with remanence of 1.23 T, (**e**,**i**) actual forces and estimated forces based on test datasets from FMFTS with remanence of 1.23 T, (**b**) actual locations and estimated locations based on test datasets from FMFTS with remanence of 1.28 T, (**f**,**j**) actual forces and estimated forces based on test datasets from FMFTS with remanence of 1.28 T; (**c**,**d**,**g**,**h**,**k**,**l**) case 2, (**c**) actual locations and estimated locations based on test datasets from FMFTS with remanence of 1.23 T, (**g**,**k**) actual forces and estimated forces based on test datasets from FMFTS with remanence of 1.23 T, (**d**) actual locations and estimated locations based on test datasets from FMFTS with remanence of 1.28 T, (**h**,**l**) actual forces and estimated forces based on test datasets from FMFTS with remanence of 1.28 T; (**m**,**n**,**q**,**r**,**u**,**v**) case 3, (**m**) actual locations and estimated locations based on test datasets from FMFTS with remanence of 1.23 T, (**q**,**u**) actual forces and estimated forces based on test datasets from FMFTS with remanence of 1.23 T, (**n**) actual locations and estimated locations based on test datasets from FMFTS with remanence of 1.28 T, (**r**,**v**) actual forces and estimated forces based on test datasets from FMFTS with remanence of 1.28 T; (**o**,**p**,**s**,**t**,**w**,**x**) case 4, (**o**) actual locations and estimated locations based on test datasets from FMFTS with remanence of 1.23 T, (**s**,**w**) actual forces and estimated forces based on test datasets from FMFTS with remanence of 1.23 T, (**p**) actual locations and estimated locations based on test datasets from FMFTS with remanence of 1.28 T, (**t**,**x**) actual forces and estimated forces based on test datasets from FMFTS with remanence of 1.28 T.

The force and location estimation results using testing datasets are shown in Figure 8, and the mean absolute error (MAE) and root mean squared error (RMSE) are summarized in Table 3. Figure 8a,b,e,f,i,j give the results for case 1, the training sets are acquired on FMFTS with remanence of 1.23 T, Figure 8a,e,i are the test results using datasets acquired from FMFTS with remanence of 1.23 T, Figure 8b,f,j are the test results using datasets acquired from FMFTS with remanence of 1.28 T; BP_ftp_1.23 in Table 3 gives MAE and RMSE in case 1. It can be seen that the mean absolute error of x, y, and F (MAE_x, MAE_y, MAE_F) for FMFTS with remanence 1.23 T are 0.1817 mm, 0.3336 mm, and 0.0144 N, respectively, while MAE_x, MAE_y, and MAE_F for FMFTS with remanence 1.28 T are 19.0919 mm, 15.5591 mm, and 3.0408 N, respectively. It is impossible to predict the contact force and location acting on the tactile sensor with remanence of 1.28 T.

Figure 8c,d,g,h,k,l give the results for case 2, the training sets are acquired on FMFTS with remanence of 1.23 T, Figure 8c,g,k are the test results using datasets acquired from FMFTS with remanence of 1.23 T, Figure 8d,h,l are the test results using datasets acquired from FMFTS with remanence of 1.28 T; BP_ptp_1.23 in Table 3 gives MAE and RMSE in case 2. It can be seen that MAE_x, MAE_y, and MAE_F for FMFTS with remanence 1.23 T are 0.3369 mm, 0.3293 mm, and 0.0179 N, respectively, while MAE_x, MAE_y, and MAE_F for FMFTS with remanence 1.28 T are 0.5675 mm, 0.6167 mm, and 0.0299 N, respectively. Compared with constructing the mapping relationship between contact force and location and magnetic flux densities in case 1, establishing the relationship between contact force and location and pose of permanent magnet in case 2 can predict contact information acting on the FMFTS with remanence of 1.23 T and 1.28 T, which shows that the robust contact force and location estimation method proposed in this paper is feasible. Furthermore, the difference in estimation error under different pressures is not large. In an ideal simulation environment, the location estimation error using the proposed algorithm is less than 1 mm, and the force error is about 0.0282 N; the accuracy may be further improved by increasing the number of training datasets.

Figure 8m,n,q,r,u,v give the results for case 3, the training sets are acquired on FMFTS with remanence of 1.23 T, Figure 8m,q,u are the test results using datasets acquired from FMFTS with remanence of 1.23 T, Figure 8n,r,v are the test results using datasets acquired from FMFTS with remanence of 1.28 T; RBF_ftp_1.23 in Table 3 gives MAE and RMSE in case 3. It can be seen that MAE_x, MAE_y, and MAE_F for FMFTS with remanence 1.23 T are 1.1853 mm, 1.2158 mm, and 0.0392 N, respectively, while MAE_x, MAE_y, and MAE_F for FMFTS with remanence 1.28 T are 886.0721 mm, 690.9536 mm, and 85.8573 N, respectively. Similarly, the contact force and location acting on the tactile sensor with remanence of 1.28 T cannot be predicted.

Figure 8o,p,s,t,w,x give the results for case 4, the training sets are acquired on FMFTS with remanence of 1.23 T, Figure 8o,s,w are the test results using datasets acquired from FMFTS with remanence of 1.23 T, Figure 8p,t,x are the test results using datasets acquired from FMFTS with remanence of 1.28 T; RBF_ptp_1.23 in Table 3 gives MAE and RMSE in case 4. It can be seen that MAE_x, MAE_y, and MAE_F for FMFTS with remanence 1.23 T are 1.3664 mm, 1.4441 mm, and 0.0565 N, respectively, while MAE_x, MAE_y, and MAE_F for FMFTS with remanence 1.28 T are 1.4633 mm, 1.7049 mm, and 0.0556 N, respectively. The RBF neural networks show similar assessment results, that is, if the relationship between contact force and location and magnetic flux densities is constructed, the neural network calibrated by the FMFTS with remanence of 1.23 T is not suitable for FMFTS with remanence of 1.28 T due to the inconsistency of the magnetic source. After constructing the relationship between contact force and location and pose of permanent magnet, the influence of the inconsistency of the magnetic source is reduced, and the neural network calibrated by the FMFTS with remanence of 1.23 T is also suitable for the FMFTS with remanence of 1.28 T. Therefore, the contact information of the FMFTS with remanence of 1.28 T can be obtained correctly.

In brief, although the performance of BP_ptp_1.23 is slightly worse than BP_ftp_1.23, in BP_ptp_1.23, the robust estimation method could estimate the contact information of FMFTS with remanence of 1.28 T, while BP_ftp_1.23 cannot. Furthermore, from the error analysis of the training results, it can be seen that the error of the test results obtained by the BP neural network is lower than the RBF neural network in this scenario. Therefore, case 2, namely the robust estimation of contact force and location for FMFTS considering magnetic source inconsistency, is feasible.

## 4. Experimental Evaluation of Robust Force and Location Estimation Method

### 4.1. Experimental Setup

**Figure 9:**
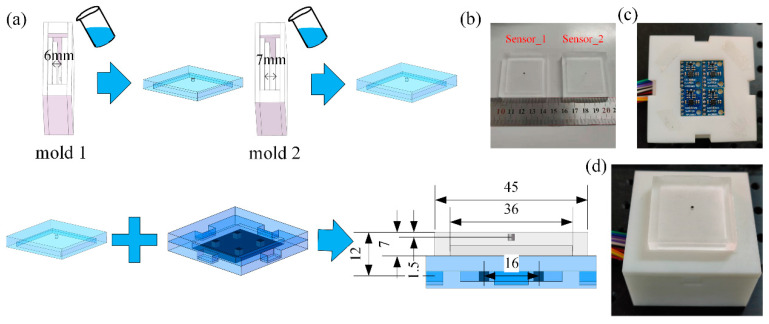
(**a**) Schematic of the fabrication process; (**b**) flexible substrate embedded with permanent magnet of different remanence; (**c**) magnetic sensor array; (**d**) flexible magnetic-based-field tactile sensor prototype.

Similar to the above simulation, we create two FMFTSs (Sensor_1 and Sensor_2) with the remanence of 1.046 T and 1.068 T and build the sensor calibration platform to verify the proposed force and location estimation method. As shown in Figure 9, the fabrication of FMFTS comprises three steps: (1) fabrication of flexible substrate; (2) fabrication of magnetic sensors; (3) assembly.

The manufacturing process of the flexible body has several stages: (1) PDMS (Sylgard 184, Dow Corning) where a ratio of elastomer to curing agent of 10:1 is prepared. After thoroughly mixing and vacuum degassing, PDMS is poured into mold 1 for vacuum degassing and curing at room temperature; (2) the upper part of the mold is taken out and the permanent magnet is placed into the remaining groove; (3) this is replace with a new mold, and the PDMS liquid is poured into the new mold 2; (4) vacuum degassing: when it is completely cured at room temperature, the shaped flexible substrate is taken out. Four magnetic sensors (Honeywell HMC5983) are used to locate the permanent magnet. Since the I2C communication address of the magnetic sensor is fixed, TCA9548a is applied to select the channel and Stm32F103 is used to collect magnet flux density data. Finally, the flexible substrate and HMC5983 are assembled.

**Figure 10:**
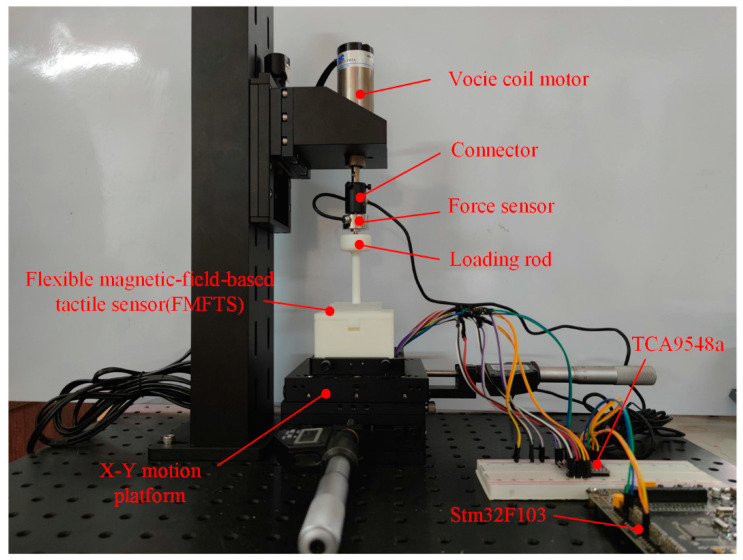
Calibration platform of tactile sensor.

As is shown in Figure 10, the calibration platform for FMFTS consists of the X–Y motion platform (DZWNX120-CD, Winner Optics, Beijing, China), voice coil motor (CBL35-010-71-1M3A, SMAC, Carlsbad, CA, USA), force sensor (LSZ-F02C, OBTE, Suzhou, China), and a 6 mm diameter loading rod. The force exerted on the tactile sensor can be controlled by the voice coil motor and force sensor, and the X–Y motion platform controls the position of the force exerted on FMFTS.

### 4.2. Experimental Procedure

The origin of the coordinate system is set at the center of the FMFTS. The external forces for FMFTS act between −14 mm and 14 mm with 2 mm intervals, and at each point, external force varies from 0 N to 4 N with 0.5 N intervals. A total of 1800 datasets were collected from Sensor_1, and the calibration was implemented based on datasets from Sensor_1; we randomly collected 90 datasets from Sensor_1 and Sensor_2 with different remanence to test the proposed method. The training data and the test data are the magnetic flux of the permanent magnet at the four three-axis magnetic sensors under different contact information, which is converted into a pose by magnetic source tracking algorithm, then the BP neural network built the mapping between contact force and location and pose of the permanent magnet, namely BP_ptp_Sensor_1.

In actual measurement, the magnetic sensor will be interfered with by external magnetic noise. Before loading the flexible magnetic-based tactile sensor, we first need to collect the magnetic noise in the environment, and then subtract the magnetic noise from the training set and test set data in order to obtain a real signal.

### 4.3. Experimental Results and Discussion

**Figure 11:**
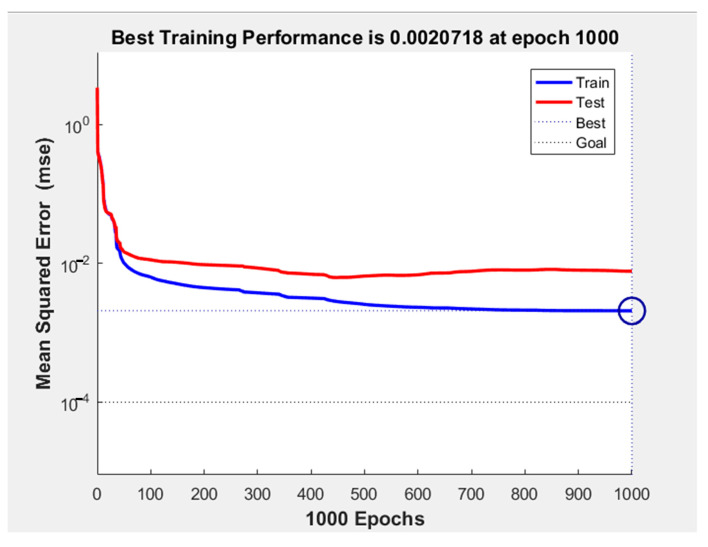
The training process of the BP neural network.

The training process of the neural network is shown in Figure 11. Mean squared error decreases with epoch and has little variation after 500 epochs.

**Figure 12:**
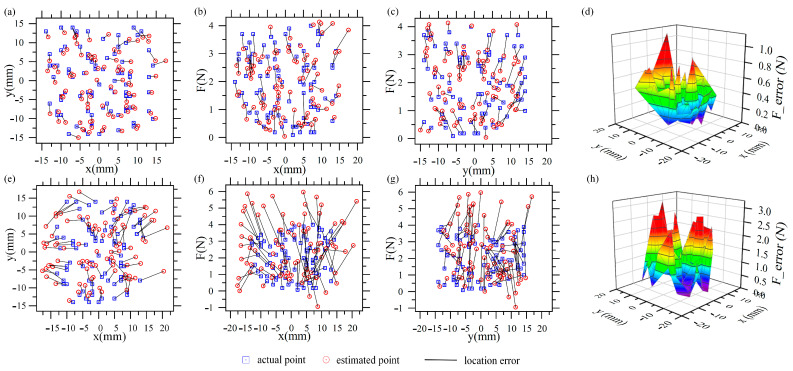
Force and location estimation results: (**a**) Actual locations and estimated locations based on test datasets from Sensor_1; (**b**,**c**) actual forces and estimated forces based on test datasets from Sensor_1; (**d**) the error of actual forces and estimated forces based on test datasets from Sensor_1; (**e**) actual locations and estimated locations based on test datasets from Sensor_2; (**f**,**g**) actual forces and estimated forces based on test datasets from Sensor_2; (**h**) the error of actual forces and estimated forces based on test datasets from Sensor_2.

The force and location experimental estimation results using testing datasets are shown in Figure 12, and the mean absolute error (MAE) and root mean squared error (RMSE) are summarized in Table 4. The training sets are acquired on Sensor_1, Figure 12a–d are the test results using datasets acquired from Sensor_1, Figure 12e–h are the test results using datasets acquired from Sensor_2; BP_ftp_Sensor_1 in Table 4 gives MAE and RMSE. It can be seen that the mean absolute error of x, y, and F of Sensor_1 are 0.7923 mm, 0.9889 mm, and 0.3385 N, respectively. The mean absolute error of x, y, and F of Sensor_2 are 2.8450 mm, 2.2251 mm, and 1.2008 N, respectively, which indicates that by using the neural network trained by Sensor_1, Sensor_2 can also estimate contact force and location. Compared with the location error, the force error is larger.

Within the 8 mm range of the length and width of Sensor_2′s surface, the mean absolute error of x, y, and F are 1.8207 mm, 1.8755 mm, and 0.7958 N, respectively, and it can be seen from Figure 12h that the error near the center is smaller. The deformation of FMFTS at the edge is small, which leads to low signal-to-noise ratio (SNR) and poor prediction results. It is also possible that the accuracy of the 3D printing mold is not high enough, resulting in differences in the mechanical properties of the tactile sensors. However, the prediction results basically meet the requirements of tactile sensing. It is possible to improve the accuracy of contact force and position estimation by proper structure design of the flexible substrate to increase SNR or by improving the production accuracy of the FMFTSs to make the FMFTS’s mechanical response consistent.

In conclusion, the mapping between contact force and location and position and orientation of magnetic source was calibrated in one FMFTS and can be used to estimate the external force and location for sensors with the same structure, which means that this estimation method of contact force and location can tolerate remanence inconsistency of magnetic source in FMFTS and improve robustness of external force and location estimation.

## 5. Conclusions

In conclusion, we have proposed an effective method for robust estimation of contact force and location for the FMFTS, considering magnetic source inconsistency. Differently to the previous research, we have improved the sensing mechanism of flexible magnetic-field-based tactile sensor. The proposed method is evaluated in simulations and experiments. The result of experiments is that the location error and force error of FMFTS (Sensor_2) without recalibration are 2 mm and 1 N, respectively, which confirms that the estimation of contact force and location for FMFTS with same structure and different remanence could reach acceptable accuracy, which depends on single calibration. This method improves the robustness between tactile sensors, and provides convenience for large-scale use and replacement of damaged tactile sensors. The tactile sensor can adjust the shape and elastic modulus of the flexible medium according to different application scenarios and achieve the purpose of predicting external contact information through the data-driven methods.

In the future, we will consider the influence of shear force, prove that this method is also feasible under shear force, and extend its application to complex situations such as multipoint contacts. When estimating the pose of the permanent magnet, we only use the LM algorithm to solve the problem; however, a dynamic tracking method such as Kalman filter or particle filter will be used to locate the permanent magnet in order to reduce the influence of magnetic noise.

## Figures and Tables

**Table 1:** Magnetic field simulation results of permanent magnets with different remanence.

	Pose_1					Pose_2				
rema(T)	1.18	1.20	1.23	1.25	1.28	1.18	1.20	1.23	1.25	1.28
p1_x_(G)	−1.3707	−1.3939	−1.4287	−1.4520	−1.4868	−1.3668	−1.3899	−1.4247	−1.4479	−1.4826
p1_y_(G)	−1.3801	−1.4035	−1.4386	−1.4620	−1.4971	−0.4252	−0.4324	−0.4432	−0.4504	−0.4612
p1_z_(G)	0.8084	0.8221	0.8427	0.8564	0.8767	−0.3668	−0.3730	−0.3823	−0.3885	−0.3978
p2_x_(G)	−1.3702	−1.3934	−1.4283	−1.4515	−1.4863	−1.3611	−1.3842	−1.4188	−1.4419	−1.4765
p2_y_(G)	1.3707	1.3939	1.4287	1.4520	1.4868	0.4183	0.4254	0.436	0.44308	0.45371
p2_z_(G)	0.8055	0.81912	0.8396	0.8533	0.8737	−0.3655	−0.3717	−0.381	−0.3872	−0.3965
p3_x_(G)	1.3844	1.4078	1.4430	1.4665	1.5017	0.8588	0.8733	0.8952	0.9097	0.93155
p3_y_(G)	−1.3796	−1.403	−1.4380	−1.4614	−1.4965	−1.5771	−1.6038	−1.6439	−1.6707	−1.7108
p3_z_(G)	0.8082	0.8219	0.8424	0.8561	0.8767	1.4492	1.4737	1.5106	1.5351	1.572
p4_x_(G)	1.3711	1.3944	1.4292	1.4525	1.4873	0.8572	0.8717	0.8935	0.908	0.9298
p4_y_(G)	1.3737	1.397	1.4319	1.4552	1.4901	1.5788	1.6055	1.6457	1.6724	1.7126
p4_z_(G)	0.8032	0.8168	0.8372	0.8508	0.8713	1.4503	1.4749	1.5117	1.5363	1.5732

**Table 2:** The calculation result of the poses of the permanent magnets.

	Pose_1					Pose_2				
rema(T)	1.18	1.20	1.23	1.25	1.28	1.18	1.20	1.23	1.25	1.28
a(mm)	−0.0313	−0.0314	−0.0312	−0.0313	−0.0314	0.5380	0.5381	0.5381	0.5382	0.5380
b(mm)	0.0443	0.0442	0.0442	0.0441	0.0444	0.0063	0.0061	0.0063	0.0063	0.0062
c(mm)	12.2578	12.2578	12.2579	12.2578	12.2579	11.7935	11.7935	11.7935	11.7934	11.7935
m	−0.0052	−0.0053	−0.0054	−0.0056	−0.0057	0.6561	0.6672	0.6838	0.6950	0.7117
n	0.0051	0.0051	0.0053	0.0053	0.0055	0.0005	0.0005	0.0005	0.0005	0.0005
p	0.9816	0.9982	1.0232	1.0398	1.0647	0.6849	0.6965	0.7139	0.7255	0.7429

**Table 3:** Error analysis of the training result of neural networks.

Train_Model	Test_re(T)	MAE_x(mm)	MAE_y(mm)	MAE_F(N)	RMSE_x(mm)	RMSE_y(mm)	RMSE_F(N)
BP_ftp_1.23	1.23	0.1817	0.3336	0.0144	0.28226	0.73234	0.0230
	1.28	19.092	15.559	3.041	22.231	21.943	4.0066
BP_ptp_1.23	1.23	0.3369	0.3293	0.0179	0.5565	0.6497	0.0280
	1.28	0.5675	0.6167	0.0299	0.8404	1.1090	0.0491
RBF_ftp_1.23	1.23	1.1853	1.2158	0.0392	1.6199	1.9133	0.0493
	1.28	886.072	690.953	85.857	906.338	743.304	86.767
RBF_ptp_1.23	1.23	1.3664	1.4441	0.0565	1.9219	2.0262	0.0859
	1.28	1.4633	1.7049	0.0556	1.9229	2.6503	0.0806

**Table 4:** The error analysis results of the experiment.

Train_Model	Test_Model	MAE_x(mm)	MAE_y(mm)	MAE_F(N)	RMSE_x(mm)	RMSE_y(mm)	RMSE_F(N)
BP_ptp Sensor_1	Sensor_1	0.7923	0.9889	0.3385	1.1293	1.3296	0.4103
	Sensor_2	2.8450	2.2251	1.2008	3.5743	2.9337	1.5005

## Data Availability

Not applicable.
